# Cardioprotective effects of triiodothyronine supplementation against ischemia reperfusion injury by preserving calcium cycling proteins in isolated rat hearts

**DOI:** 10.3892/etm.2020.9581

**Published:** 2020-12-16

**Authors:** Lichao Fang, Zhiping Xu, Jian Lu, Lei Hong, Shigang Qiao, Lijun Liu, Jianzhong An

Exp Ther Med 18:4935–4941, 2019; DOI: 10.3892/etm.2019.8114

Subsequently to the publication of the above article, an interested reader drew to the authors’ attention that the GAPDH panel of the western blots shown in Figs. 2A and 3A appeared to be remarkably similar to the PMCA data panel shown in Fig. 4A, albeit at different exposures.

The authors have reviewed the original data, and realized that, during the preparation of the figures submitted in the original manuscript, incorrect data were selected for the PMCA data panel in Fig. 4A. Consequently, a corrected version of Fig. 4 is shown opposite, containing the correct data for the PMCA experiment.

Note that this error did not affect the overall conclusions reported in the paper. The authors sincerely apologize for the error caused by the inadvertent selection of incorrect data in Fig. 4A, and thank the reader for drawing this error to their attention. The authors also apologize to the Editor of *Experimental and Therapeutic Medicine* and to the readership for any inconvenience caused.

## Figures and Tables

**Figure 4:**
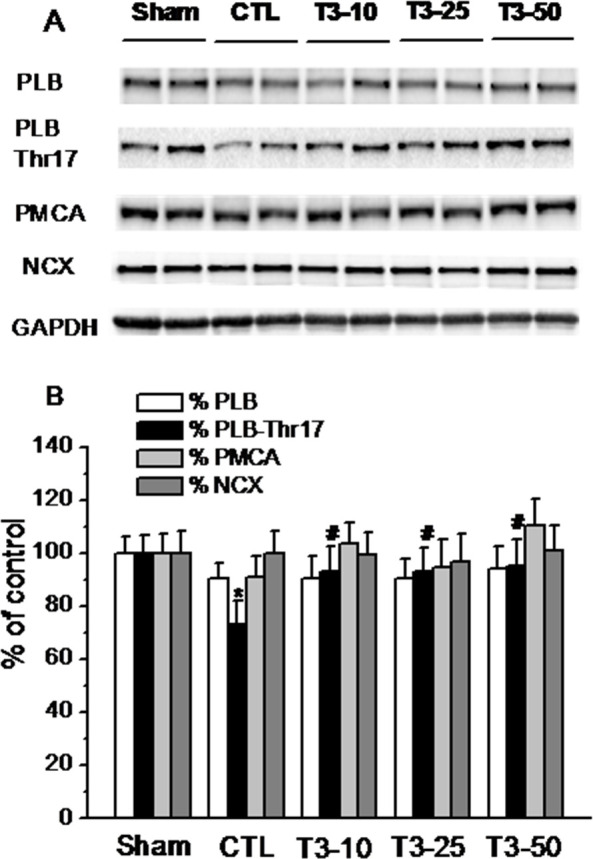
Western blot analysis of PLB and its phosphorylation at Thr-17 (PLB-Thr-17), PMCA and NCX in the homogenate of myocardial tissue from rat hearts. The data are presented as the percentage of controls. (A) Western blots presenting the levels of PLB, PLB-Thr-17, PMCA and NCX protein. Bands are quantitative immunoblots of representative samples from the hearts of the different groups run on the same gel. (B) Group results. Values (mean ± standard deviation) in the treatment groups are expressed as the percentage of the control group. ^*^P<0.05 vs. time control group (Sham); ^#^P<0.05 vs. the CTL group. PLB, phospholamban; PMCA, plasma membrane Ca^2+^-adenosine triphosphatase; NCX, sodium-calcium exchanger; CTL, ischemia control; T3, Triiodothyronine.

